# Fresh funds for moms: feasibility of a 12-week online food as medicine grocery prescription program for women with food insecurity and gestational diabetes

**DOI:** 10.3389/frhs.2025.1625558

**Published:** 2025-09-03

**Authors:** Rachel Gillespie, Sara J. Maksi, Joshua Bush, Cynthia Cockerham, Courtney T. Luecking, Andrea L. Deierlein, Heather Wasser, Alison Gustafson

**Affiliations:** ^1^ Department of Health, Behavior and Society, College of Public Health, University of Kentucky, Lexington, KY, United States; ^2^ Department of Dietetics and Human Nutrition, Martin Gatton College of Agriculture, Food, and Environment, University of Kentucky, Lexington, KY, United States; ^3^ Kentucky Injury Prevention Research Center, University of Kentucky, Lexington, KY, United States; ^4^ Department of Obstetrics & Gynecology, Division of Maternal Fetal Medicine, College of Medicine, University of Kentucky, Lexington, KY, United States; ^5^ Public Health Nutrition, School of Global Public Health, New York University, New York, NY, United States; ^6^ Department of Nutrition, Gillings Schools of Global Public Health, University of North Carolina at Chapel Hill, Chapel Hill, NC, United States

**Keywords:** food as medicine, gestational diabetes, type 2 diabetes, hypertension, food insecurity, grocery prescription, online grocery shopping

## Abstract

**Introduction:**

Pregnant women with food insecurity experience high rates of gestational diabetes mellitus (GDM). Food as medicine and grocery prescription (GPx) programs have been successful in increasing food access and managing chronic disease; however, they are often not implemented during pregnancy.

**Methods:**

This was a pilot study of Fresh Funds for Moms, an online grocery prescription (GPx) program. Pregnant women between 20 and 28 weeks' gestation were purposefully recruited from one large hospital system in an urban community in Kentucky. Eligibility included: positive screening for food insecurity; diagnosis of either GDM, type 2 diabetes, or hypertension; and live in a zip code with online grocery service delivery access. Women received $200 per month for 12 weeks (total of $600) for healthy food purchases on an online grocery platform.

**Results:**

A total of 1,163 women were initially screened; 20 women were referred to participate in the program and 14 completed the program. On average, women redeemed 96.1% of their grocery benefit throughout the pilot GPx program. Fruit and vegetable purchases increased 4% from months one to three (27%–31%), while the percentage of funds spent on meat food items decreased from 31% to 22% by the third month, and percentage spent on pantry items remained consistent month-to-month (4%). Qualitative findings highlight overall satisfaction, however, participants reported some transactional confusion when redeeming their funds on the online grocery platform and a desire for more variety when selecting food items for delivery. Blood glucose and blood pressure improved slightly, however no clinically meaningful changes in HgbA1c were observed.

**Discussion:**

This pilot study demonstrated the feasibility of implementing a GPx program in collaboration with clinical, research, and industry partners as a food as medicine intervention. Online GPx programs have the potential for improving healthy shopping habits among pregnant women. However, to improve screening, referral, and enrollment, a larger system approach is needed to meet patients' needs, warranting further investigation in larger, adequately powered studies.

## 1 Introduction

Food as Medicine (FAM) and Grocery Prescription (GPx) intervention programs have increased in popularity and interest among healthcare systems and clinicians over the last decade as a potentially more sustainable and viable approach to improving chronic disease management through nutrition intervention (1, 2). Despite advancements in understanding of clinical management of chronic diseases, such as cardiovascular disease and type 2 diabetes, the challenge remains for patients to effectively implement nutritional recommendations, a burden that is especially felt among those experiencing food insecurity (3). These programs address the issue of access to the healthy foods meeting condition specific needs, while also addressing nutrition insecurity, which is defined by the United States Department of Agriculture (USDA) as “consistent and equitable access to healthy, safe, affordable foods essential to optimal health and well-being” (4). A novel application of FAM and GPx is for the management of gestational diabetes mellitus (GDM), which increases the risk of pregnancy complications and has lasting impacts on health outcomes for both mother and infant (5).

GDM impacts approximately 5%–10% of pregnancies in the United States (6); defined as the new onset of hyperglycemia in pregnancy and typically diagnosed between 20 and 28 weeks gestation, depending on when screening takes place (7, 8). GDM is associated with an increased risk of pregnancy complications that contributes to $1.6 billion in health care costs annually (9, 10). The health impacts of GDM on mothers and infants are not limited to the prenatal period. Approximately 50% of pregnant women who develop GDM will develop type 2 diabetes (6). Additionally, GDM increases the risk of cardiovascular events in the decade following pregnancy (11). Infants born to mothers with GDM carry a lifetime increased risk for obesity and diabetes, reinforcing the importance of intervening during this critical period in order to improve long-term health outcomes (5, 12).

Food security in the United States has become a fundamental component of the social determinants of health (SDOH) and is broadly defined as consistent and reliable means of access to enough food that supports an active, healthy lifestyle (4). However, the SDOH are multi-faceted, and when fractured can lead to poorer outcomes. This is exacerbated for individuals with low income and racial or ethnic minorities, who display a higher risk for developing GDM, further propagating health disparities within these populations (13). Among adults, food insecurity is linked to decreased nutrient intakes, increased rates of mental health problems and depression, increased rates of diabetes, cardiometabolic risk, and complications from diabetes (14–18). A study from 2021 reported that 7% of pregnancies were complicated by diabetes and 86% of those women had GDM (19). A key factor in the rise is inadequate access to food in pregnancy and reported food insecurity (13). The lack of adequate nutrition not only affects the mother's health, but it can also limit the ability to manage other health conditions during pregnancy, specifically diabetes, since food insecurity worsens an individual's ability to implement nutritional recommendations to effectively manage blood glucose (20). The culmination has lasting implications on fetal growth and development (21), which has elevated food insecurity as a leading healthcare issue. Existing food assistance programs (i.e., SNAP and WIC) serve an important role in reducing food insecurity and improving diet adequacy for pregnant women (22, 23). However, these programs were designed to improve general diet adequacy and quality rather than address condition specific nutritional needs.

GPx programs, also referred to as medically-tailored grocery programs, apply a FAM lens to food assistance, where the “food package” becomes a “prescription” aimed at managing or treating a specific clinical condition (24). The goal of these programs is to address both issues of food security as well as nutrition security by providing tailored healthy food interventions aimed at improving clinical outcomes. Recent reports from Aspen Institute indicate that these programs are highly successful in improving health outcomes (25). However, key implementation factors have yet to be identified. To scale implementation of FAM programs, a clear understanding of the organizational-level, individual-level, and innovation-level factors that could facilitate or be a barrier to integration is needed. Implementation science frameworks, such as the Consolidated Framework for Implementation Research (CFIR), can guide evaluation of barriers and facilitators to direct innovation adaptations and inform strategies to promote scaled and sustained implementation of FAM initiatives.

Methodology for implementing GPx programs varies but primarily focuses on fruit and vegetable provision and research on these programs to address nutritional needs in pregnancy is limited. In a recent systematic review of GPx programs (2), one study included pregnant women, which provided four $40 monthly farmer's market vouchers and only 56% of the women redeemed at least one voucher (26). One of the barriers to redeeming the vouchers was proximity to a farmer's market. Utilizing online grocery ordering and delivery has the potential to reduce barriers around procuring healthy foods, such as time, travel, and ease of use of the program. Further, grocery home delivery services are generally widely available and have been successfully used to improve access to healthier foods (27, 28). Locher et al. piloted grocery delivery services in young mothers participating in WIC, finding that delivery services were feasible and participants felt that the service improved their ability to make health habits (27). Incorporating behavioral nudge strategies and promoting health-centric choice architecture has also been shown to optimize behavior changes (29). This has increasingly been an area of focus as online grocery shopping use has increased and geographically expanded. When accessibility barriers are then coupled with health promoting strategies to “nudge”, such as individualized text messages, short and long term behavior change can be achieved among target populations.

In the current pilot study, we evaluated the feasibility for implementation of a GPx and home food delivery program among a high-risk pregnant population, including identifying eligible patients, screening and recruitment efforts, referral to the pilot program, and enrollment in the study through partnership with clinical staff, study team members, and food-delivery industry partners.

## 2 Materials and methods

Fresh Funds for Moms was a 12-week online GPx program, paired with weekly text messages, for high-risk pregnant women with food insecurity, providing $200 per month for healthy food purchases and delivery from an online grocery platform. The study took place from August 2023 to April 2024 created through a partnership between an academic research institution and a large university hospital system network located in Lexington, Kentucky, and a food-delivery online grocery shopping platform service (30). Study protocol was reviewed and approved by the University of Kentucky IRB (protocol #86500) and registered at ClinicalTrials.gov (Fresh Carts for Mom's to Improve Food Security and Glucose Management- Identifier: NCT05979519).

### 2.1 Framework

The Consolidated Framework for Implementation Research (CFIR) is one of the most widely utilized guiding determinant frameworks within and outside the realm of implementation science (31). CFIR outlines five domains (Intervention/Innovation Characteristics, Outer Setting, Inner Setting, Characteristics of Individuals, and Process) and provides a structured lens to identify key determinants that may support or hinder successful implementation within a defined setting or context (32). In this study, we utilized a modified CFIR to understand finite characteristics as a way to identify and inform the key implementation process factors currently operating as barriers or facilitators for implementation. Individuals serve as the central figure engaging with the GPx program (the Intervention/Innovation domain) although in different capacities. Additionally, we focus on the characteristics of the implementation process to analyze the specific factors involved at screening, referral, and enrollment delivery points for this FAM approach.

To examine the CFIR implementation factors and characteristics of the Fresh Funds for Mom's GPx program, we focus on 1. individual characteristics (providers and patients) related to the uptake of the GPx program through qualitative response feedback and quantitative measures including: a. screening metrics; b. referral and enrollment rates; and c. program engagement metrics. Second, 2. program characteristics (Intervention/Innovation domain) by assessing the purchasing and redemption patterns of participants throughout the 12-week period; and 3. implementation process characteristics which will be captured through process measures from clinic staff involved in the screening and referral process and from individuals who enrolled in the program. The combination of these engagement measures overall provides insights into key aspects for how to approach screening, referral, enrollment, and engagement of a FAM program based on the CFIR framework.

### 2.2 Study participant screening, referral, and enrollment

Prospective participants were pregnant women who were receiving prenatal care at the University of Kentucky Healthcare (UKHC) Obstetrics and Gynecology (OBGYN) clinic and were scheduled for a prenatal scan between August 2023 and January 2024. Eligible women were first identified by nursing staff using the following criteria: gestational age between 20 and 28 weeks; documented high risk pregnancy, defined as a pregnancy complicated by any condition that poses an actual or potential risk to the health of the mother or fetus (33); and diagnosis of GDM, T2DM, or hypertension (HTN, added for eligibility in November 2023) documented in the electronic medical record (EMR). Since screening for food insecurity had not been fully implemented and automated into the EMR system at this point [for more extensive methodology and context see Mayfield et al. (30)], women who met these criteria were then manually screened by nursing staff using the two-question Hunger Vital Signs food security questions (34). Food insecurity was defined as an affirmative response (“often true” or “sometimes true”) to one of the two questions:

“Within the past 12 months, we worried whether our food would run out before we got money to buy more.”

“Within the past 12 months, the food we bought just did not last as we did not have money to get more.”

**Figure 1:**
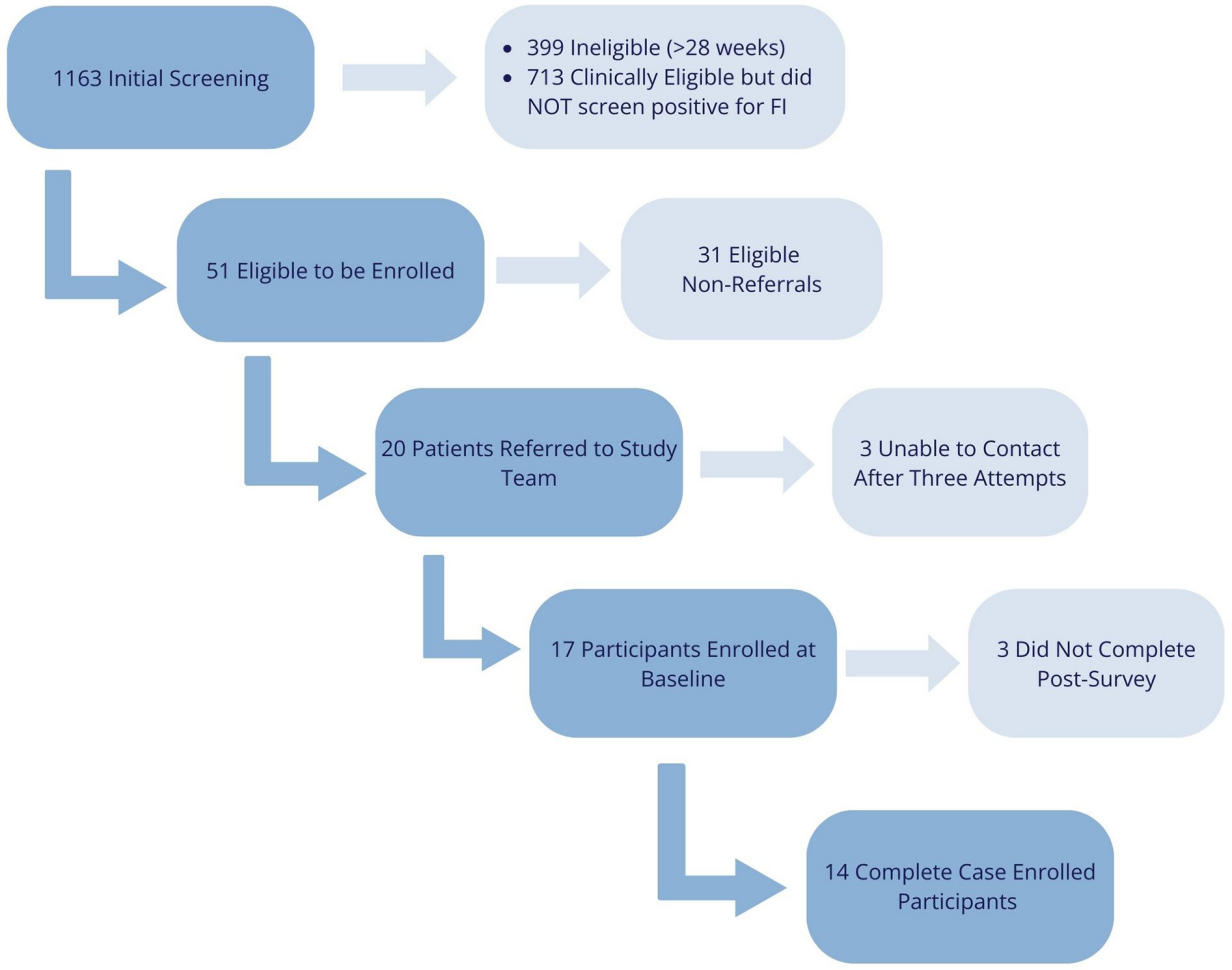
Screening, referral, and enrollment study flow diagram for the fresh funds for moms program.

Women who met all eligibility criteria were entered into a REDCap project shared with study staff (*n* = 20) (35, 36). The women were sent a text message and email inviting them to participate in the Fresh Funds for Moms project, including a brief description and link to an eConsent document with demographic questions. Three follow-up text and/or email attempts were sent in an effort to initiate and continue the referral and enrollment processes. Zip code was collected as part of the demographic questions to confirm eligibility for grocery delivery through the online GPx program. Women outside the delivery area were not eligible to participate, while those in eligible zip codes were enrolled and completed a baseline survey. Figure 1 outlines the complete screening, referral, and enrollment rates of women into the Fresh Funds for Moms program. Participants received a $25 gift card as compensation for completing the baseline survey.

### 2.3 Online GPx program

Food items included in the Fresh Funds for Moms GPx program were based on the American Diabetes Association plate method, which has been shown to improve ease of creating healthy meals and contribute to management of blood glucose levels (37). To simplify identification and shopping for participants, eligible items were individually denoted on the grocery platform with a Fresh Funds “tag” next to the product—similarly how an item on sale may be distinguished on a digital interface. Food items included: all fruits and vegetables (dried, frozen, fresh, and canned); protein foods (chicken turkey, eggs, fish, shellfish, lean beef cuts such as round sirloin, flank, and tenderloin, pork chops and pork tenderloin, lean deli meats of ham and turkey, cheese, and cottage cheese); plant based protein (beans, lentils, hummus, nuts, nut butters, edamame, tofu and tempeh, and plant-based meat substitutes); and dairy products (unflavored milk, yogurt, milk substitutes like soy and almond milk). We also included bottled water, since water insecurity is often associated with food insecurity and has been demonstrated to undermine human health and development (38). The following food items were excluded/ineligible for purchase: pantry items, including oils and condiments; juices, due to variability in sugar content; cream cheese, butter and dairy spreads, sour cream, yogurt drinks or pouches, and prepared foods (e.g., deli items, frozen entrees) due to variability in nutrient content; pasta, due to inability to differentiate whole grain from other varieties; and soups, because they were not part of the Diabetes Plate program.

Since enrollment occurred on a rolling basis, the onboarding process to the GPx program and online platform typically began within 24 h of completing the consent form and baseline survey. Participants received a series of text messages sent directly by a study team member guiding them through set up and activation of their online grocery service account. Each participant received a direct link with an individualized unique code to add to their online grocery shopping account and activated the GPx program period (12-weeks). Once confirmed participants were directed to the online grocery platform's Fresh Funds for Moms “storefront” to begin their grocery delivery order. The storefront consisted of a curated “Fresh Funds for Moms” program page, which displayed all eligible GPx grocery items based on availability within the participant's geographic area. These storefronts leveraged choice architecture elements and provided a user-friendly mobile grocery shopping environment catered to the participants in the program. Orders could be submitted directly from the storefront or participants could navigate to the online grocery platform's standard storefront to view and purchase items not included in the GPx program. However, purchasing any non-eligible GPx items required the participant to use a personal payment method to place and complete the order. Participants also had the option of adding and applying other qualifying monthly stipends to their online grocery service account, such as Supplemental Nutrition Assistance Program (SNAP) or WIC benefits, that could be used at checkout when submitting online grocery orders.

Participants received 1–2 text messages per week throughout the study period that were scheduled in advance to automatically send as part of the behavioral “nudge” text message component of the program. Simultaneous and individualized text messaging has shown to be effective health promotion strategy to encourage short-term behavior change (39, 40), and has previously been a successful approach utilized by the study team (29). The messages included periodic redirects to the storefront, reminder texts about placing orders with funds, and self-efficacy driven messaging. One example of a text message read: “*Achieving your goals can be the most satisfying feeling & can be rewarding when you save money & time by doing your grocery shopping online with your Fresh Funds. Staying motivated & on track with your health can help keep you and baby healthy, strong, & fed. Keep up the great work*!”

### 2.4 Measures

Data was drawn from the participant baseline and post-program surveys, purchasing data provided by the online grocery service, and biometric data derived from the EMR. The baseline survey captured self-reported responses to individual and adult household food security levels (41), and measured self-efficacy levels related to clinical self-management (e.g., “I am able to check my blood sugar or blood pressure if necessary”) and dietary habits (e.g., “I am able to follow a healthy eating plan when I am away from home”). At the conclusion of the 12 weeks, a text was sent with a REDCap survey link to complete a post survey, with the same questions from the baseline survey and open-ended questions to provide feedback on their experiences and overall satisfaction with the program. See Supplementary Materials for all a posteriori responses collected from participants. Those that completed the post survey received a $25 gift card as compensation.

A total of $215.96 per month for three months was applied to each participant's online grocery shopping account as part of the online GPx program. The additional $15.96 was applied automatically as needed by the online grocery platform towards any taxes and service or delivery fees that were incurred as participants completed online grocery orders. This amount was predetermined by the online grocery partners and participants maintained $200 of buying power each month as part of their enrollment in the online GPx program. Any remaining unused funds did not carry over from previous months and automatically reset within 1–3 business days for months two and three. Purchase reports were sent monthly to the study team from the online grocery industry partners that included the Gross Merchandise Values (GMV), defined as the value associated with a given order item (includes item cost, taxes, and fees); and a list of all food items that had been purchased with each unique code in the GPx program. Food items were classified by levels (L1–L3) increasing in specificity: L1) food, beverage; L2) Fruit/Vegetable (F/V), Deli, Dairy, Pantry, Meat, Eggs/Other, No Calorie Beverage; L3) Fresh F/V, Canned F/V, Frozen F/V, Milk/Cheese, Eggs, Deli Meat, Fresh Meat Cuts, Snacks, Other Protein, Grains.

Study team members met in July 2023 to determine appropriate biometrics to be collected from participants at baseline and at 12-weeks (or the last recorded values prior to delivery). All biometric values were abstracted from participants' EMR by the clinical nursing staff and entered into REDCap. If a participant delivered prior to the end of the 12-weeks, they continued to receive the Fresh Funds to supplement grocery purchases and postpartum grocery purchase data was not used. Final biometric data included: blood pressure (BP), maternal weight (wt), height (ht), number of weeks gestation, fasting blood glucose (BG), and hemoglobin A1c (HgbA1c).

### 2.5 Data analysis

For analyses, purchases were grouped either by participant-month (i.e., first, second, or third month of participation) or total purchases during the program by level (i.e., L1, L2, or L3). The Gross Merchandise Value (GMV) was used to quantify purchases and in some cases exceeded the value of the voucher due to promotional prices. The statistical model included number of meals as a covariant to control and appropriately adjust for anyone who completed the 12-weeks at postpartum. Descriptive statistics were conducted using SPSS (Version 29) to summarize study sample characteristics. Biometric data was not tested for significance due to the small sample size and because of the nature of this feasibility study and the process objectives described above. Short answers provided in the post-survey (Supplementary Material) were analyzed by qualitative description and were used for report-back and evaluation purposes with the online grocery platform partners (42, 43).

## 3 Results

A total of *n* = 14 women were successfully screened, referred, enrolled, and completed the 12-week Fresh Funds for Mom's GPx program along with the post-survey. The program period, which began once the participant had been fully enrolled and onboarded, spanned from October 2023 to April 2024. All zip codes where referred patients (*n* = 20) reported living were able to be served. Table 1 provides descriptive characteristics of the participants that completed the program. The average age of the women at baseline (time point 1) was 29 (range 20–40 years old) and 23-weeks gestation.

**Table 1:** Demographics of *n* = 14 participants that completed the “fresh funds for moms” online GPx program.

Descriptive	*N* (%)
Race/Ethnicity	
White	10 (71.4%)
Black/African American	3 (21.4%)
Hispanic, Latinx/e, or Spanish	1 (7.1%)
Household Income	
<$15,000	3 (21.4%)
$15,000–$29,999	4 (28.6%)
$30,000–$44,999	3 (21.4%)
$45,000–$74,000	3 (21.4%)
>$75,000	1 (7.1%)
Education	
Some High School	1 (7.1%)
High School Graduate/GED	4 (28.6%)
Some College	1 (7.1%)
Vocational or Trade School or Program	2 (14.3%)
Associate's Degree	2 (14.3%)
Bachelor's Degree	3 (21.4%)
Graduate or Professional Degree	1 (7.1%)
Employment Status	
Full-Time	8 (57.1%)
Part-Time (economic reasons, not by choice)	1 (7.1%)
Unemployed	3 (21.4%)
Disabled	2 (14.3%)
Food Nutrition Assistance Programs^a^	
SNAP^b^	4 (28.6%)
WIC^b^	7 (50.0%)
Food Pantry	1 (7.1%)
None	4 (28.6%)
Government Assistance Programs^b^	
TANF^b^	1 (5.9%)
Medicaid	8 (57.1%)
None	5 (35.7%)

^a^ Respondents able to select more than one answer choice (“select all that apply”).

^b^ SNAP, supplemental nutrition assistance program; WIC, women infants children; TANF, temporary assistance for needy families or other cash assistance. .

Complete screening, referral, enrollment, and execution process phases, and their associated quantitative outcome values, of the Fresh Funds for Moms project are described in Table 2. These process measures are comprehensive in nature and therefore report on findings from the entire duration of the project. Of note, the most extensive phases include the screening and referral steps, which encompass (and required) several points of service and procedures prior to enrollment and engagement in the GPx program. This was identified as the most significant implementation process barrier for the individuals engaging with the FAM program within the clinical/healthcare setting due to the administrative burden and capacity restrictions.

**Table 2:** Process measures for a GPx program delivered through a healthcare referral system.

Healthcare delivery service point	Process measure	Definition	Individuals (*n*=)
Screening		Initial screening of all UKHC patients scheduled for OBGYN anatomy scan between August 2023 and January 2024	1,163
	Ineligible	Patients outside gestational age window (≥28^0^)^a^	399
		Patients with documented high-risk pregnancy + met clinical criteria, but did not screen positive for food insecurity.	713
	Eligible to be Enrolled	Patients meeting all inclusion and eligibility criteria.	51
	Eligible Non-Referrals	Patients that fell outside eligible gestational age window (>28 weeks) due to extended communication during screening process—unable to be referred.	31
Referral	Referral Rates	(20/51)	39%
	Eligible Patients Approached for Enrollment	Referred to study team—Clinical staff added patient to REDCap Project	20
	Eligible Non-Participants	Study team unable to contact after three attempts to continue enrollment.	3
Enrollment	Enrollment Rate	(17/20)	85%
	Total Participants Enrolled at Baseline	Participants that completed consent + pre-survey	17
	Clinical Dx of those Enrolled	GDM alone	4
		T2DM alone	6
		HTN alone	3
		Multiple Dx (combination of T2DM & HTN)	4
Connection to Resources (i.e., Intervention Retention)	Complete Case Total Participants	Individuals that completed full program (pre- and post-surveys + purchasing) and all intervention components.	14
		Enrollees that completed pre-survey but NOT post-survey	3
		Opted out of texts at some point during the 12-weeks	8
	Completion Rate	(14/17)	82%
Impact on Purchasing Outcomes	Percentage of Food Categories Purchased with All GPx Dollars for All Participants During 12-Week Intervention Period	Fruits/Vegetables	27%
		Deli	7%
		Dairy	20%
		Pantry	4%
		Meat	28%
		Eggs/Other	13%
		No Calorie Beverages	2%
	Gross Merchandise Value (GMV) of Purchases by Participants with GPx Dollars During 12-Week Intervention Period	Fruits/Vegetables	$2,252.24
		Deli	$589.03
		Dairy	$1,672.89
		Pantry	$311.10
		Meat	$2,404.81
		Eggs/Other	$1,097.90
		No Calorie Beverages	$145.77

^a^ 28^0^ = Indicates 28 weeks and 0 days, patient outside gestational age window of eligibility.

Based on the total order amount, which included fees associated with each order, participants expended 96.1% of the available funds during the 3-month program period. Half of participants used more than 99% of the funds and only one participant used less than 90% of the funds (82.4% was the minimum expenditure). The average number of orders per participant during the program period was 6.7 orders, equivalent to approximately 2 orders per month, with a range of 3 (1/month) to 15 (5/month) orders. Using the GMV, which does not include order fees, participants received groceries valued at 100.9% of the allocated funds. GMV in some cases exceeded the purchase amount due to promotional pricing on items.

**Figure 2:**
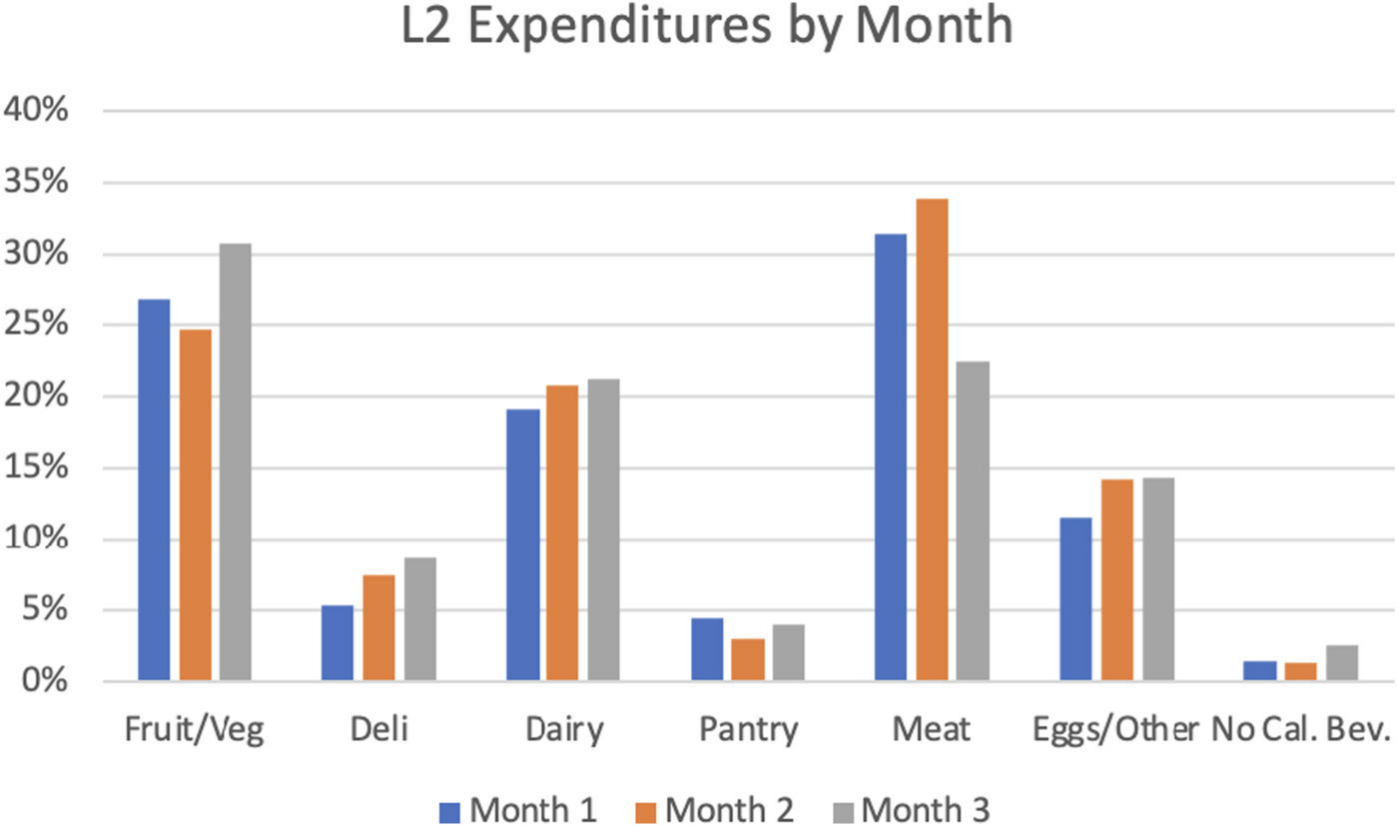
Percentage of fresh fund dollars spent on each food category by participant each month.

Purchases categorized at L1 revealed that a very small percentage of the GPx funds were allocated to beverages (2%) while the remainder was spent on food items. L2 further differentiated between food types and showed that spending was highest on meat (28%), fruit and vegetables (27%), and dairy (20%). The most granular level of reporting was L3 where meats were reported as fresh cuts (28%) or deli (8%) and fruits and vegetables were separated into fresh (19%), canned (4%), and frozen (4%). Between months one to three, there was an increase in overall spending for fruits and vegetables (+4%), deli (+4%), dairy (+2%), and eggs and other protein (+2%) food items, while purchases of meat dropped (−10%). See Figure 2.

Only one woman delivered prior to the end of the program. All other participants who completed the program (*n* = 13) were pregnant for the duration of the 12-week period. At baseline, mean (SD) gestational age was 23 weeks (2.4) and women had average weight of 243 lbs (51.5) and BMI of 39 kg/m^2^ (8.2). Blood glucose levels slightly decreased during the study period (97 mg/dl to 94 mg/dl), as did blood pressure (121/80 mmHg to 118/79 mmHg). The HgbA1c values remained clinically unchanged (6.2%–6.4%). Table 3 shows the biometric measures for participants at baseline and after completing the 12-week GPx program.

**Table 3:** Biometric outcome measures among participants (*n* = 14) in the fresh funds for moms GPx program pre- and post-intervention (12-weeks).

Biometric measure	Baseline (Time Point 1) Mean (SD)	Post-intervention (Time Point 2) Mean (SD)
Weeks Gestation	23 (2.6)	35 (2.3)
Height (inches)	65.6 (2.2)	65.6 (2.2)
BMI (kg/m^2^)	39 (8.8)	40 (8.6)
Weight (kg)	108.4 (25.5)	112.4 (24.5)
Weight (lbs)	242.9 (56.1)	257.9 (58.2)
Blood Glucose (mg/dl)	97 (10.2)	94 (14.9)
Blood Pressure (mmHg)	121/80 (11.4/7.6)	118/79 (10.1/6.9)
HgbA1c (%)	6.2 (1.5)	6.4 (1.5)

## 4 Discussion

This pilot study aimed to evaluate the feasibility of implementing a GPx program for high-risk pregnant women, a novel population. Specifically, we aimed to identify the key points in which patient screening and referral systems bottleneck between healthcare organizations and community or industry partners, and address health-related social needs efficiently in a multi-dimensional capacity. The purchasing patterns displayed here are consistent with other findings that revealed engaging in online grocery shopping led to healthier purchasing habits over time (44). The Fresh Funds for Moms online GPx program provided the link between offering financial assistance and a diverse selection of medically tailored grocery items. This allowed the women to exhibit personal preferences and maintain agency when placing orders, while continuing to promote the purchase of healthy foods and beverages—exemplifying libertarian paternalism through this approach to behavior change (45). By month three, women were spending nearly a third of their monthly funds on fruits and vegetables—the bulk of which was spent on fresh produce compared to canned or frozen. This reinforces the idea that provisioning an online GPx program could lead to improved dietary adherence (1), which would be beneficial for addressing numerous clinical conditions in addition to targeting food insecurity. Interestingly, the direct feedback participants shared at the conclusion of the program (Supplementary Material) was relatively in line with purchasing patterns, including the affinity for purchasing produce and meat products. However, considering not every participant provided feedback since sharing responses was not a requirement to complete the post-survey, incorporating mixed-method study designs would be beneficial as programs such as these are refined to ensure potential changes remain user-focused. Slight improvements in fasting blood glucose and blood pressure were observed, however no clinically meaningful changes in A1c within the small sample we enrolled. More studies are needed to accurately determine any long-term changes on clinical outcomes, including whether food items purchased are actually consumed. These small enrollment numbers were largely due to the logistical challenges encountered throughout the screening process. While clinical biomarkers were not a main outcome measure to determine program effectiveness, it is still a promising indicator that these types of programs could be effective if logistical issues can be addressed and bolsters the need for additional, larger sample size, and longitudinal studies that are appropriately powered.

Further, this study demonstrates the complexities of the screening and referral systems that are currently in place within the healthcare structure. At the time this study took place, food insecurity (FI) was not routinely screened for, despite this and other social determinants of health (SDOH) being a frequent predictor for some clinical outcomes (46–48). The step of manually conducting the food insecurity screening for each eligible patient posed a significant burden on the clinical staff. Since clinic staff capacity was limited, this hindered the referral process considerably—and thus, enrollment—into the study. Furthermore, because of the lag-time that resulted from the manual screening, 31 patients that ultimately were eligible to be referred for enrollment fell outside the gestational age eligibility window (over 28 weeks) due to delayed response times from patients or clinic staff. This component drastically extended the time and effort required for the screening process and subsequently had the largest impact on the referral rate. Furthermore/additionally, the timing of GDM diagnoses identified to be a key clinical parameter that should be considered when enrolling in future. More specifically, GDM diagnoses were occurring later while T2DM diagnoses were identified earlier among this cohort of high-risk pregnant women, which may have contributed to the loss of eligible patients referred to the study team during the screening and enrollment phases. In the future, enrollment should extend to 32 weeks to carry women into post-partum, given these complexities and challenges associated with screening and referral. Extending program duration overall to carry women through delivery and into post-partum (i.e., two weeks after giving birth) would be a critical study design component advocates and researchers should consider prioritizing. However, as FI and other SDOH screenings become more common and integrated into the clinical screening process standard of care (49), there is potential for the identification of vulnerable patients to be more efficient. This systemic approach could lead to improved capacity building by developing the necessary infrastructure that addresses the connection between social needs and health outcomes.

The enrollment and onboarding phases of the project revealed keen insights to consider for future implementation of GPx programs using food delivery services. Overall, we had success utilizing automated text messages and emails to invite those referred to enroll and to initiate the onboarding process. This helped address the gap in time between when screening and connection to resources occurred, which has been noted as a frequent challenge for healthcare organizations (50). Communication regarding the user experience with the online grocery shopping platform throughout the duration of the Fresh Funds for Mom's project was typically shared via text message with the study team. These communications facilitated onboarding and initial engagement; however, platform-specific troubleshooting was limited. Therefore, the technological logistics required to translate these types of programs and initiatives to scale, may not necessarily be feasible at this time. Additionally, relying on these forms of technology and communication alone is not sufficient, given nearly half of the participants we enrolled opted out of text message communications (i.e., responded “STOP” to the reminder/program campaign text messages) at some point throughout the 12-week period. This is an important consideration when evaluating overall participant engagement with this GPx program, particularly since there is strong literature supporting the instrumental efficacy of behavioral nudges and improving chronic disease self-management (51). Although this study did not directly capture participant motivations to unsubscribe, the team did capture participant's lack of enthusiasm for receiving text messages over the study period by eliciting a “STOP” option.

One novel component of this project was the utilization of an online grocery shopping platform that provided home food delivery services. Although online grocery shopping may help address challenges with procurement and logistics to consistently purchase healthy food items, utilizing an online shopping platform may not be appropriate for all populations. This could include those that may be unbanked (do not have access to or utilize traditional financial institutions or services, including possessing a savings/checking account, credit or debit cards, or personal checks) since inputting a credit or debit card was a requirement for online grocery platform interface; or those residing in more rural communities. However, it is important to note that despite these technological barriers, redemption rates of program funds were consistently high across all participants for each of the months enrolled in the program. Leveraging technology for reachability serves a critical role in addressing existing food access barriers (i.e., transportation access), however, infrastructural broadband expansion is still required for many rural communities to support the necessary wifi connectivity these forms of technology require. Scalable reproducibility may be limited as all functions of the online grocery shopping platform may not be available for certain participants and/or customer bases as a result.

Several critical points have been ascertained and should be considered for future scalable work in this space. Findings from this pilot study should be reassuring for policymakers and researchers when considering implementing other large, pragmatic randomized trial designs—such as the KP ENRICH trial currently underway (52)—and forecasting similar outcomes. However, a fundamental component of focusing on real-world effectiveness is simultaneously developing or identifying feasible, smooth, and sustainable processes for all stakeholders, which this study successfully revealed to facilitate for future efforts. This pilot revealed key insights for participant engagement and clinical staff burden, which are paramount factors in determining the success of initiatives or programs.

## 5 Conclusion

Although this pilot did not produce participant enrollment numbers that would be powered to detect any effects from the GPx program, our results offer promising insight as to the feasibility of conducting screening, referral, and enrollment into a FAM type of program with clinical, research, and industry partners with this population. Further, showcasing certain purchasing patterns offer support to the effectiveness of this type of program on alleviating food insecurity and producing a positive effect on blood pressure and blood glucose levels—both of which could improve certain clinical outcomes—such as the ones studied here. Considering produce (fruits and vegetables) were the second most ordered food items among this demographic, coupled with the feedback shared by participants and the specific elements to retain, GPx programs delivered in this capacity could be a reasonable dissemination approach to support patients in a more wholistic and sustainable way. This should be considered in the future with larger scale studies adequately powered with respect to identifying effective multilevel approaches that carry the potential to produce a meaningful impact on patient physical health.

## Data Availability

The raw data supporting the conclusions of this article will be made available by the authors, without undue reservation.
