# Supplementary material for: Fresh funds for moms: feasibility of a 12-week online food as medicine grocery prescription program for women with food insecurity and gestational diabetes
Source: Front Health Serv. 2025 Sep 3;5:1625558. doi: 10.3389/frhs.2025.1625558 (PMC12440972; doi:10.3389/frhs.2025.1625558)
Supplement: Supplementary file 1 [file Table1.docx]

Supplementary Material

# Participant Open Ended Post-Survey Feedback with the Fresh Funds for Moms Program and Potential Barriers to Consider in Future

| ***Survey Questions^1^*** | | | |
| --- | --- | --- | --- |
| **What would you change about the Fresh Funds for Mom’s program?** | **What would you keep about the Fresh Funds for Mom’s program?** | **What was confusing or challenging about your experience with the Fresh Funds for Mom’s program, if anything?** | **Were there any food items you wanted to use Fresh Funds for Mom’s for that you couldn't? Please describe what that was.** |
| ***A Posteriori Participant Responses ^1^*** | | | |
| More food options | The delivery option | Which stores I could use. local stores didn't always show as available. | Ground beef |
|  | The ability to order online and it come right to your door |  | Bacon |
| I didn't enjoy using the delivery part of the program because I live rurally. multiple stores more locally to me did not appear as available on my app so delivery people had to come farther from farther stores even though there were closer options. | Loved the fresh funds label to pick healthy foods. Appreciated the extra money to give me more options/availability to healthy foods. | What was applicable and what wasn't (ex. Different flavors of the same yogurt) | Ground beef wasn't available but chunked beef was? |
|  |  |  | Yes, some meats weren't approved |
| Have more applicable foods covered - example multiple brands of the same cheese | Variety of items included | Being able to see how much you got left to spend! | More variety of granola bars & cereal, more meat products |
| I might would add a little more types of meat | To keep getting it in the future | Understanding how much my card would be charged at the end of purchases, it would often reflect a different price | Milk, bread, peanut butter |
|  | The amount that they give, it's a great amount! |  | Hamburger, water & juice |
| Allowing purchase of water or drink related items | Everything, it was extremely helpful |  | Cased water, provided food but no drinks |
|  | I love everything about the program |  | Water, ground beef, breads, frozen meats |
| Understanding that delivery would cost, and add more of a selection of food items, and duration of program | The produce selection |  | Breakfast meats were generally not covered |
| ^1^ Questions were not required to complete, thus, not all participants provided comments on all/any of the open-ended questions to be reported here. All responses provided have been included. | | | |
